# Bio-electrochemical frameworks governing microbial fuel cell performance: technical bottlenecks and proposed solutions

**DOI:** 10.1039/d1ra08487a

**Published:** 2022-02-16

**Authors:** Rehab H. Mahmoud, Ola M. Gomaa, Rabeay Y. A. Hassan

**Affiliations:** Water Pollution Research Department, National Research Centre (NRC) Dokki Giza Egypt; Microbiology Department, National Center for Radiation Research and Technology (NCRRT), Egyptian Atomic Energy Authority (EAEA) Nasr City Cairo Egypt olagomaa4@gmail.com; Nanoscience Program, University of Science and Technology (UST), Zewail City of Science and Technology 6th October City Giza 12578 Egypt; Applied Organic Chemistry Department, National Research Centre (NRC) Dokki 12622 Giza Egypt

## Abstract

Microbial fuel cells (MFCs) are recognized as a future technology with a unique ability to exploit metabolic activities of living microorganisms for simultaneous conversion of chemical energy into electrical energy. This technology holds the promise to offer sustained innovations and continuous development towards many different applications and value-added production that extends beyond electricity generation, such as water desalination, wastewater treatment, heavy metal removal, bio-hydrogen production, volatile fatty acid production and biosensors. Despite these advantages, MFCs still face technical challenges in terms of low power and current density, limiting their use to powering only small-scale devices. Description of some of these challenges and their proposed solutions is demanded if MFCs are applied on a large or commercial scale. On the other hand, the slow oxygen reduction process (ORR) in the cathodic compartment is a major roadblock in the commercialization of fuel cells for energy conversion. Thus, the scope of this review article addresses the main technical challenges of MFC operation and provides different practical approaches based on different attempts reported over the years.

## Abstract 2

Sustainable operation requires addressing key MFC-bottleneck issues. Enhancing extracellular electron transfer is the key to elevated MFC performance.

## Graphical abstract

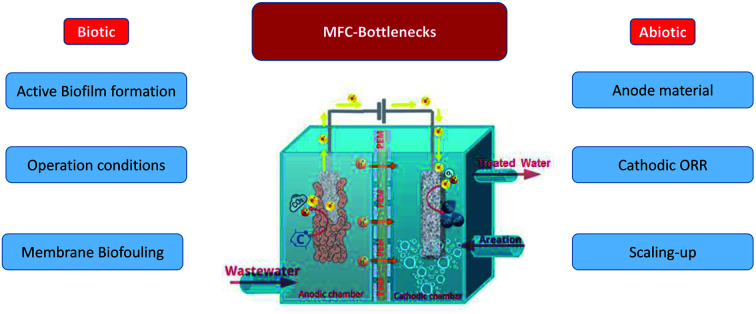

## 1. Introduction

Microbial fuel cells (MFCs) are bio-electrochemical systems that exploit living-microorganism metabolites to generate an electrical current through the oxidation of a variety of degradable organic substances.^1^ In addition, anaerobic microbes can obtain electrons from the oxidation of inorganic electron donors such as sulfide.^2^ This concept of producing electricity using microbes was first published by M. C. Potter in 1911 (ref. 3) using *Escherichia coli*. After a series of developments, especially from 1990 onwards, the power output of MFCs improved with a reduction in the component and operating costs.^4,5^ Recently, MFCs have been used as power sources for miniature and lab scale electronic apparatus.^6^ Recognizable improvement has also been achieved for wastewater treatment, water desalination, biohydrogen production, and biosensors by MFCs.^7–10^ However, to date scale-up and commercialization of MFCs aren't economically feasible, mainly owing to their low stability, high operating cost and low current generation.^11^ This review article is dedicated to providing the readers, both students and professionals, with an overall view and thorough discussion of the main limiting factors of MFC operation and suggestions are made to improve performance.

## 2. Functioning of the microbial fuel cell (MFC)

**Fig. 1:**
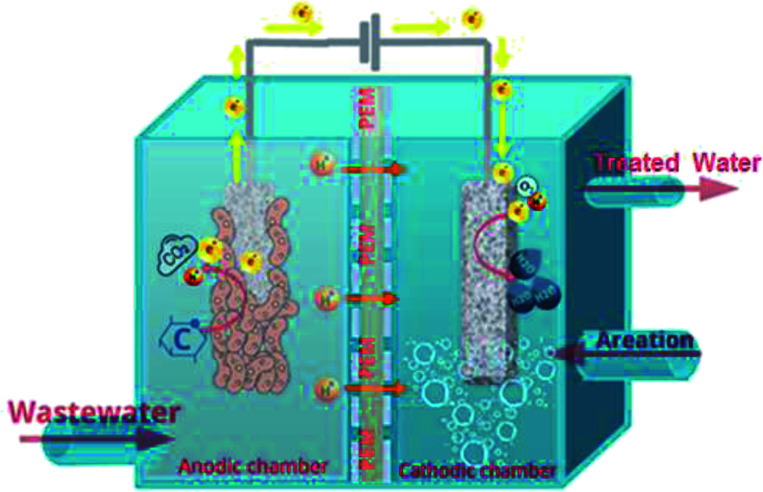
A schematic representation of a double-chamber microbial fuel cell (MFC) device showing the cathodic and anodic compartments separated by a proton exchange membrane (PEM).

An MFC is a bioelectrochemical system that produces electricity by converting chemical energy stored in the organic substrates to useful electrical energy by utilizing the catalytic activities of microorganisms.^12^ Mainly, the function of an MFC can be visualized from the schematic of a basic configuration of MFC presented in Fig. 1. Several other novel and potentially effective configurations of MFCs have been developed, regardless, the main function, as described below, remains constant for any given MFC configuration. The basic MFC design consists of an anode and a cathode separated by a proton exchange membrane (PEM). For the anodic reaction, living exoelectrogenic or electroactive organisms catalyze the breakdown of organic degradable matter (electron donors) that are transferred from the bulk solution *via* the cellular metabolism to the anodic surface with the generation of electrons.^13^ Convenient electrocatalysts of biological origin complete the reduction reactions at the cathode, where the protons transported through the PEM are combined with electrons and oxygen to form water molecules. It is confirmed that electrons could be liberated from any biodegradable substrate, varying from pure fuels including acetate, glucose, cysteine, ethanol, and bovine serum albumin to complex mixtures of organic substances such as domestic, animal, meat-packing, and food-processing wastewaters.^14^

## 3. Microbe-electrode synergy

**Fig. 2:**
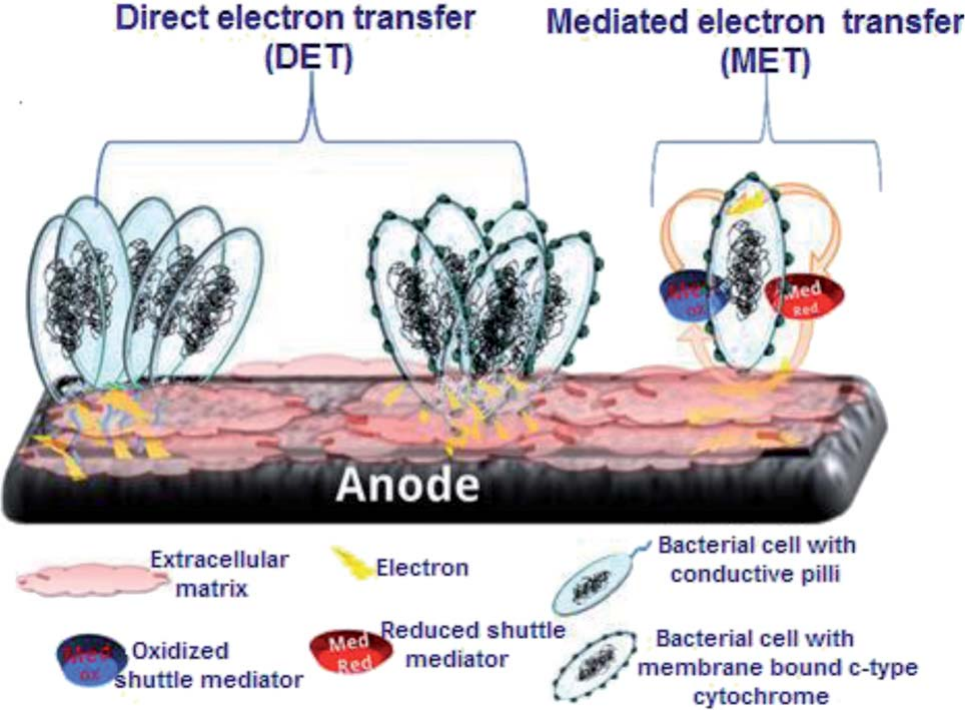
Illustration of the extracellular electron transfer in the microbial electrochemical systems. The direct microbial electron transfer (DET) is enabled through the growing of conductive pili, or by membrane-bound c-type cytochrome on the microbial cell wall. The mediated electron transfer (MET) was conducted either *via* the reduced shuttle mediators or secretion of electroactive secondary metabolite(s).

One of the most important key aspects of MFC technology lies in the mechanism by which microorganisms adhere to the electrode surface and the subsequent electron transfer that takes place. Since electrodes are surfaces that cannot enter the bacterial cells, the main requisite is that electrons are to be transferred from the inside living microbial cell membrane to the outer membrane either through the physical transfer of reduced compounds or *via* electron bouncing across the membrane using membrane-bound redox enzymes.^15^ Regardless of the mechanism, the extracellular electron transfer must result in a redox-active species that can link the bacterial cell to the electrode electronically. This species may be a soluble redox shuttle, a reduced primary metabolite, or an outer membrane redox protein.^15^ To this date, several extracellular electron transfer (EET) mechanisms between living microorganisms and electrodes have been proposed. As shown in Fig. 2, two different mechanisms can be used for connecting the living microbial cells with the electrode surface through direct electron transfer (DET) or mediated electron transfer (MET). The full description of each mechanism is given in the next section.

### 3.1. Direct electron transfer (DET)

The DET mechanism is dependent on the ability of electroactive bacteria, also known as exoelectrogens to transfer electrons extracellularly to conductive materials directly without mediators *via* anodic respiration.^16,17^ DET which occurs through the physical contact between the bacterial cell membrane or a membrane organelle and the electrode surface^18^ can be carried out by bacterial membrane-redox-active proteins (c-type cytochromes).^19–22^ Likewise, some microbes can produce conductive nanowires, which represent a modified form of DET.^23,24^

For example, *Shewanella oneidensis* MR-1 produces conductive filaments that are extensions of the outer membrane, thus they allow electrons flow from the cell membrane to a terminal electron acceptor.^25–28^*Geobacter sulfurreducens* also produces a type-IV pili which is capable of electrical conductivity *via* overlapping pi–pi orbitals that exist in aromatic amino acids.^29^ DET is not limited to bacteria, a wide range of microbes were also reported as potential exoelectrogenic due to evidence of DET mechanism. This has been explained by a mediator-less bioelectrochemical approach for studying the intracellular level of *Candida albicans*.^30,31^ Lately, several studies started screening various algal species for their exoelectrogenic activity and the probability of direct electron transfer. One of the most interesting findings was that after achieving suitable growth conditions with the absence of light and inorganic nutrients which resulted in a significant decrease in the photosynthetic oxygen production, the blue-green alga *Oscillatoria agardhii* was able to transfer electrons directly without adding artificial redox mediators.^32^

### 3.2. Mediated-MFCs (MMFCs) challenges

Some microbes are electrochemically inactive and require soluble chemical redox mediators to transfer their electrons to the electrode surfaces.^33,34^ In order to enhance the rate of the electron transfer, many attempts were made to overcome the kinetic, and thermodynamic obstacles. Several synthetic electron mediators have been commonly used to transfer the electrons by replacing the oxygen to accept the electrons during the microbial respiration chain between the microbe and electrode, which is called mediated electron transfer.^35^ There are two types of artificial mediators that are widely used, *i.e.*, hydrophilic mediators (such as potassium ferricyanide) and lipophilic mediators such as benzoquinone, menadione, dichloroindophenol, 2,3,5,6-tetramethylphenylenediamine. Synthetic dyes can also act as mediators in MFC, in that essence neutral red was reported to enhance mediated electron transfer and increased the longevity of MFC performance as compared to MFC without neutral red.^36^ Hydrophilic mediators are unable to move across the cell membrane and are limited to reacting with the proteins located on the periplasm, however, they have the advantages of high water solubility and high diffusion coefficient in the aquatic systems, which enhance microbial fuel cells operation.^37,38^ On the other hand, lipophilic mediators such as menadione are able to permeate across the cell membrane, interacting with the intracellular redox centers in cytoplasm and mitochondria, being decreased by the intracellular enzymes, and diffusing outside the cell to transfer electrons to the electrode surface. Nonetheless, using a lipophilic mediator as a sole mediator in the MFC may not be a good choice, because their poor aqueous solubility can significantly affect their concentration in the detection system and hence impact the amplitude of the current signals. To resolve the aforementioned issue, a double-mediator system composed of a hydrophilic and a lipophilic mediator will compensate for their shortcomings and allows the intracellular redox processes, expressing intracellular cell metabolism activities of target cells and high current signal intensity.^38^ The potassium ferricyanide–menadione double-mediator system is widely applied to analyze the redox activity of *S. cerevisiae* yeast cells and mammalian cells. Moreover, this double-mediator system has been used to investigate a specific enzyme activity or biochemical process in yeast cells,^39,40^ single-cell imaging using scanning electrochemical microscopy (SECM),^41^ and the application in MFCs,^42^*etc.* Even though combining mediators can dramatically increase the current magnitude and thus boost the MFC efficiency, there are significant disadvantages. When microbes are continuously provided with supplementary electron shuttles, it is not easy to save energy for activities such as cell growth and maintenance, which is very important when considering risks for industrial MFC systems.^35^ In addition to that, synthetic soluble mediators can be leached out in flow systems for practical use, increasing the expense of the process and being unfriendly in terms of environmental protections. To address this limitation, mediators were immobilised on electrodes using different chemical methods. However, due to the reduced mobility (or permeability) of immobilised mediators, this technique was expected to inhibit the mediated electron transfer rate between microbes and electrodes.^43^ Furthermore, the costs of incorporating artificial mediators into continuous, commercial-scale processes would increase operation costs. Moreover, artificial mediators are introduced into the ecosystem, they can have unanticipated implications for human health and other living organisms.^44^

### 3.3. Self-mediator secretion

Microorganisms usually adapt to harsh environmental conditions of nutrient limitation or presence of xenobiotics. They resort to a multiplex of stress response strategies to overcome the stressful condition. For example, in conditions where biofilms are extremely dense and DET mechanisms are limited due to spatial constraints, microbes secrete water-soluble electroactive metabolite(s) in the extracellular matrix as an alternative mechanism to promote self-mediation of electron transport.^15,45^ The two microbes that have received the most attention in terms of self-mediator secretion are *Shewanella oneidensis* and *Pseudomonas aeruginosa.* The Gram-negative microbe, *S. oneidensis*, is able to secrete various mediators like flavin mononucleotide (FMN) and riboflavin.^46–49^ Marsili *et al.* reported that up to 75% of the extracellular electron transfer (EET) in *S. oneidensis* is self-mediated.^50^*S. oneidensis* mutants deficient in secreting flavins (because of deletion of bfe gene) showed a decrease in the current output when its bio-electrochemical performance was compared with the wild strain.^51,52^*P. aeruginosa*, an opportunistic Gram-negative pathogen, is the second most studied microbe for MET. Pure cultures of *P. aeruginosa* KRP1 isolated from anode biofilms established MET *via* the secretion of phenazines, as the quorum sensing (QS) molecules, in particular, pyocyanin and phenazine-1-carboxamide.^46,53^ Under aerobic conditions, pyocyanin is believed to be active in lipid and carbohydrate degradation as well as electron transfer to oxygen as a terminal electron acceptor.^54^ Even then, anaerobic condition studies indicated that pyocyanin is able to interact with iron as a terminal electron acceptor.^55^ Exploiting this kind of mediator self-producing microorganisms to run MFC is a good alternative to artificial mediators, but it necessitates the isolation and analysis of a large number of microbes that are capable of producing endogenous mediators.

## 4. Characteristics of electroactive biofilms

The biofilm formed by electrogenic microbes is known as the “powerhouse” of MFCs because it serves as the source of electrons to the electrodes for the functioning of MFCs.^56^ Biofilms are communities of bacteria that assemble to form a single or diverse organism(s) performing various metabolic activities.^56^ The existence of the electrocatalytically active anode surface is an important factor to remember when forming a matured electrochemical active biofilm from mixed culture.^57–60^ There are many techniques available to determine when an electrochemically active biofilm has been formed, including electrochemical methods such as cyclic voltammetry (CV), electrochemical impedance spectroscopy (EIS),^61^ microscopy, biological analysis, and Raman spectroscopy. Some of these techniques have drawbacks in that they often damage the biofilms or require laboratory conditions that are harmful to microbial cells. CV studies revealed the direct electrode interaction of Fe(iii) reducing bacteria, *e.g. Shewanella putrefaciens*, is only enabled when the cells are incubated with the working electrode under anaerobic conditions, whereas the electrode serves as the sole electron acceptor.^20,62^ As a result, a quasi-reversible CV with a defined cathodic peak at −0.32 V and an anodic peak at 0.03 V against a saturated calomel electrode (SCE) were obtained.^62^ Redox peaks cannot be found in CV voltammograms in the presence of oxygen since oxygen is an electron acceptor. These findings indicated that, as long as anaerobic conditions were persevered, direct electron exchange using electroactive living organisms would be possible. As a result, the DET was then investigated using a mediator-less-MFC, and substantial current output was observed when the dissolved oxygen content was decreased in the anodic chamber.^63,64^ Enrichment of a single strain from a consortium of living cultures, accompanied by biofilm formation, could lead to the discovery of novel electrochemically active isolated species from mediator-less microbial fuel cells.

**Fig. 3:**
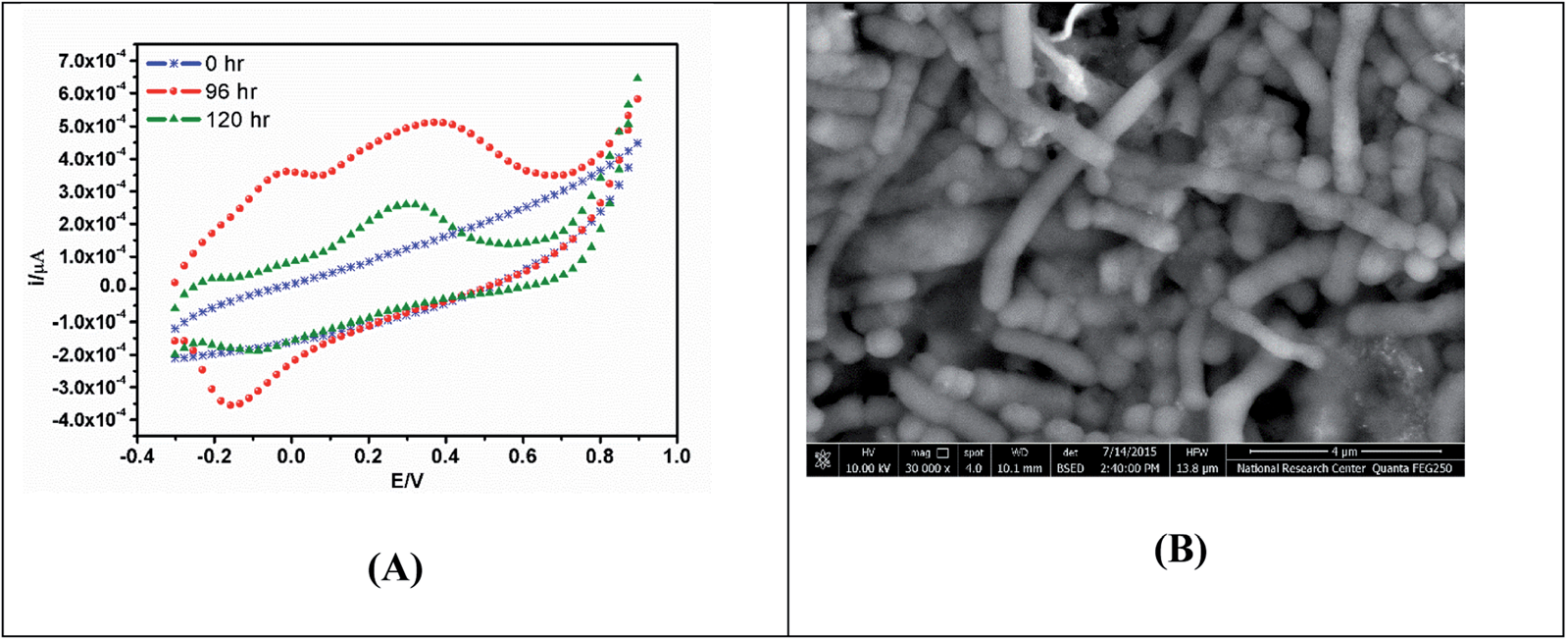
(A) CV showing bio-electrochemical responses of biofilm formation of bacterial mixed cultures grown on the MnO_2_-modified screen printed electrode for 120 h under anaerobic condition. (B) SEM image of the modified screen printed electrode (SPE) incubated in bacterial mixed cultures.^68^

Voltammetric technique can be run in real-time without requiring extensive preparation of the reactor vessel and are non-invasive; nonetheless, it changes the microbial environment for a brief period, which can influence microbial behaviour and its state during that period and potentially for a short time afterward. CV can be used coupled with other physical characterization techniques such as scanning electron microscopy, to image and visualize the mature biofilm formation at the electrode surface as shown in Fig. 3A and B.

SEM creates a 3D image and detects extracellular polymeric substances (EPS) in biofilms using scattering and absorption of electrons on the biofilm surface.^65^ On the other hand, SEM is a time-consuming technique that necessitates the fixation, and dehydration of each sample, which can damage biofilms and collapse EPS structures.^66,67^ Cryo-SEM, environmental-SEM, and focused ion beam-SEM (FIB-SEM) are SEM modifications that mostly skip the disruptive sample preparation steps but result in lower resolution images.^66^ The downside of many of these techniques is that the biofilms cease to be viable during imaging and it is difficult to see the working nature of an electro-active biofilm. To address these drawbacks, microscopy is often used in conjunction with other techniques in ambient conditions to provide real-time data on biofilm conditions.

## 5. Challenges and factors affecting electroactive biofilm formation

One of the most complicated issues in achieving high-power densities with electroactive biofilms is tackling the substrate's mass transfer limitations.^69^ Furthermore, conservation of viable bacterial cells in biofilms with effective wash out of cell debris harness the ability of electroactive biofilms. Research should be focussed on preservation methods for mixed cultures present in electroactive biofilms so that revival of the biofilm result in similar and reproducible results. Biofilm formation in microbial fuel cells is usually affected by several factors such as the electrode materials, reactor configuration, operating conditions, and biological parameters.^70^

### 5.1. The influence of MFC configuration on biofilm formation

The structural components of MFCs, such as electrode material, electrode spacing, membrane type, electrical circuit conductivity, working volume, *etc.*, not only influence biofilm formation, but also the final MFCs performance. As a result, the majority of research focus on improving MFC architecture, and testing new materials.^71,72^

#### 5.1.1. Effect of electrode materials on biofilm formation

The electrode material is critical in biofilm formation because it serves as the scaffold on which the biofilm grows and exchanges electrons to perform anodic respiration. The surface features of bioelectrodes, such as morphological structures, porosity, hydrophobicity, conductivity, charge, and bio-compatibility, depend mainly on the material used, and these properties have a direct impact on the microbial adhesion, and cell viability mechanism.^70^ The electrode's porosity contributes to an increase in the surface area, which creates more space for living microorganisms to attach and colonize. Electroactive microbes were found to be more selective towards the positively charged and hydrophilic electrode surfaces for the formation of conductive biofilms.^73^ Generally, bacteria have a net negative charge, therefore, positive charged electrodes are preferred since they provide a good electrostatic surface for negatively charged bacteria to attach to electrode surface. This attachment takes place *via* hydrogen bonding.^73^ Furthermore, electrode materials must be corrosion-resistant, have a high specific surface area and electrical conductivity, in addition to low electrical resistance and cost. The anode should be fabricated from a chemically stable material capable of operating in an environment where a wide range of organic and inorganic constituents are present. Those constituents may react with anode materials and result in poor MFC performance.^74^ Anode material should also be biocompatible to ensure viable growth and efficient electron transfer.^75^

One of the previous reports commented on the effect of electrode nanostructures on the selective capture ability of exoelectrogens from wastewater samples for enhancing the formation of electrochemically active biofilms. Accordingly, the electrochemical activities of the matured biofilm formed on the MnO_2_ modified electrode confirmed the direct electron transfer, whereas the outer redox species were involved in the extracellular electron transfer.^68^

##### 5.1.1.1. Conventional anode materials used in MFCs

**Fig. 4:**
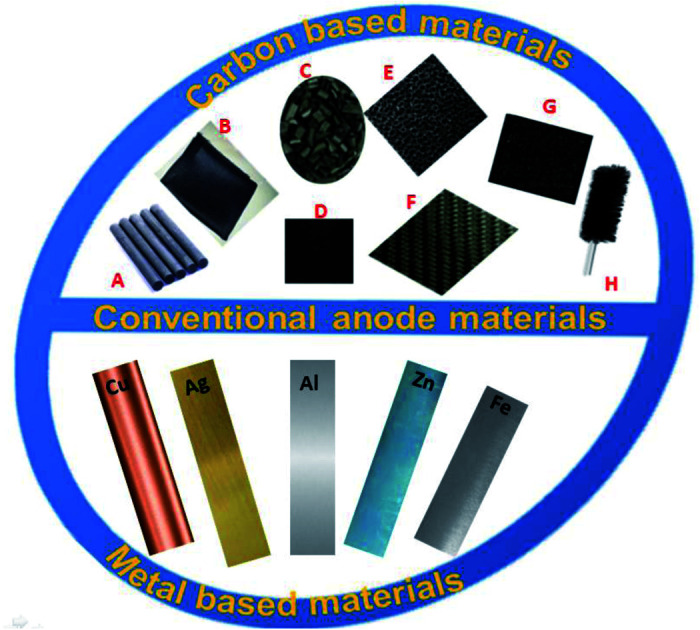
Some of the most conventional anode materials, in the upper half (A) graphite rods, (B) carbon cloth, (C) granular graphite, (D) carbon paper, (E) reticulated glassy carbon, (F) carbon mesh, (G) carbon felt, (H) carbon brush, and few metallic anode strips in the lower half.

In previous decades, numerous different materials have been investigated as anodes for MFCs. While the majority of previous research focused on the use of carbon-based materials and metal/metal oxides.^68,76–78^ Over years, these conventional materials were phased out in favor of more advanced materials that showed efficient MFC performance. The conventional carbon-based materials, shown in Fig. 4, include carbon paper (CP), carbon cloth (CC), graphite rod (GR), flexible graphite sheets (FGS), graphite felt (GF), graphite granules (GG), carbon brush (CB), reticulated glassy carbon (RGC), and activated carbon (AC).^79–82^

Both packed and plane structural configurations, as well as brushes-based conventional anodes, have all been thoroughly reviewed.^80^ Low cost is one of the benefits of using carbon-based anode materials besides biocompatibility, good electrical conductivity, and excellent chemical stability.^80^ However, some aspects limit their applications in MFCs. For instance, the graphite-based materials have strong mechanical stability, but a weak biofilm formation on the anode surface, which has a direct negative impact on the MFC performance. The CP, CC, and RGC anode have a considerable rough surface area, which is required for biofilm formation nevertheless, all these are mechanically poor and result in inefficient operation over the long-term, and repeated life-cycles. In terms of microbial adhesion, the carbon felt (CF) showed excellent conductivity and flexibility. Unfortunately, the biofilm growth prevented the diffusion of organic substrate from the exterior to the interior surface, which is unfavorable for stable bacterial colonization. This is because of the high thickness surface of CF material. On other hand, using the CBs as anode enhanced the MFC performance, but they required metal wires to fix the carbon brushes, making them financially unsuitable. This has prompted researchers to develop modified anodes using CP, CF, or GR.^77^

In addition, carbon-based materials are known for their high hydrophobicity which does not favor microbial adhesion, this results in low electron transfer capacity.^83^ Electrochemical oxidation, chemical, and heat treatment can all be used to increase the surface area of an anode and therefore enhance the adhesion of microorganisms and the output power of the microbial fuel cell.^84–87^

Several types of metal/metal oxide nanoparticles (NPs) such as MnO_2_,^68,88^ titanium,^81^ gold,^89,90^ and copper^91^ have been contemplated for use as anode catalysts in MFCs. The idea of using different metals as MFC anode has failed due to several reasons such as cost, corrosion, and non-biocompatibility.^92^ For example, stainless steel mesh meets the required criteria of an ideal anode, but due to the gravity effect, biofilm vanishes over long-term use.^93^ Likewise, while Au, Ag, and Cu have excellent conductivity, they have poor operational stability when used to produce energy over long periods. As a result, modern anode materials must be integrated into the design while searching for feasible ways to obtain them in order to achieve satisfactory MFC efficiency.

##### 5.1.1.2. Strategies of advanced anode materials

Advanced anode materials are further classified into two categories, each of which has shown their utility in both energy generation and wastewater treatment. Natural biomass-based carbon anode materials and composite-based materials, which comprise metal/metal oxides, carbon-based materials, and conducting polymers, are two subcategories of advanced anode materials.^94^ Advanced materials are expected to provide a low-cost alternative to traditional anode materials, with the added bonus of superior characteristics. The utility of advanced electrode materials used in MFCs has been the subject of numerous investigations. These advanced materials are described in the sub-sections that follow.

##### 5.1.1.3. Natural anode electrodes

Recently, manufacturing of anode electrodes from natural materials, such as biomass wastes, has gained great interest recently due to various advantages, including the use of recyclable materials, material availability and material stability.^95,96^ Natural waste-derived materials are the most effective materials for manufacturing electrodes for MFCs because they are less expensive than traditional materials and are characterized with ideal electrode features. Moreover, the micro/mesoporous 3D structure of natural waste derived materials exhibited faster electron transfer and a good electro-kinetics mechanisms for electrochemical processes in MFCs.^97^ Manufactured 3D sponge was used as low-cost and high-performance anode material for MFC.^98^ Another example is the development of layered corrugated carbon (LCC)-based anode electrodes from low-cost materials *via* carbonization. As a result, increasing the number of layers improves current density performance by providing bacteria with a larger surface area for biofilm growth. In previous studies, LCC was used as a cost-effective and high-performance anode material in MFCs. LCC-based MFCs produced about 200 A m^−2^ and 390 A m^−2^ of current density by increasing the number of layers to three and six, respectively. Their work reported that LCC enhanced the current density compared to traditional graphite felt.^96^ Almond shells, waste paper, forestry residues, wood-derived wastes, chestnut shells, corn straw, and silk cocoon were among the used natural wastes for anode electrode fabrication. The abundance of biomass waste is dependent on availability in a specific geographical area, in Malaysia, for example, palm oil-based biomass is the most readily available biomass waste.^99^ However, several recyclable-waste materials have yet to be tested for anode fabrication. Furthermore, none of the previously developed anodes from natural wastes had a sufficient conductivity efficiency to meet the modern energy demand for MFCs.

##### 5.1.1.4. Conductive polymers

Conducting polymers can be used as a sole or doping electrode materials for MFC operations due to their high conductivity and resistance to environmental conditions. The conductive polymer-modified anodes can be prepared by modification of electrode materials for enhanced bacterial cell attachment. Moreover, for improving anodic performance, the conducting polymer can be doped with nanomaterials forming a composite. Recently, many researches focused on the anode modifications with semiconductor polymers like polyaniline (PANI), polydopamine (PDA), and polypyrroles (PPy). These polymers increase capacitive properties, biocompatibility, and an active surface of the anode.^100^ When the CV method was used for doped electrochemically polymerized PANI onto graphite felt electrode,^101^ it was found that graphite felt-PANI decreased the start-up time, because the PANI-modified anode improved the bacterial cell adherence.

Anodes modified with polymers or co-modification with other metal or carbon compounds, pure cultures of *E. coli*^102^ or *Shewanella* sp*.*^103,104^ are most often used as MFC exoelectrogens. There are few studies on the impact of anode modifications with polymers on the multispecies microbial community of anode. They showed that the modifications increased the number of Proteobacteria, *Deltaproteobacteria*, and the genus *Geobacter*.^105,106^ The presence of polydiallyldimethylammonium (PDDA) on the CF electrode accelerated the adherence of exoelectrogens to the surface through electrostatic attraction. *Geobacter* sp. and *Pseudomonas* sp. were about nine and three-fold higher, respectively, on PDDA modified CF anode than on the unmodified anode.^106^ Exoelectrogens from genera *Acinetobacter*, *Brucella*, and *Bacillus* exhibited 1.4 times lower adherence on PDDA modified CC anode than on the unmodified anode.^107^ Chen and Wang^108^ showed that *E. coli* cells grown in PDDA microcarriers had the same viability as those grown in suspension, as shown by an increase in optical density and cell number. However, *Chlorella vulgaris* cells showed extremely poor viability inside PDDA microcarriers, possibly due to blockage of nutrient uptake by the diallyldimethylammonium quaternary ammonium cation.^109,110^ At anodes modified with 50% PDA, an approximate two-fold increase in the percentage of *Proteobacteria* (up to 33%) and *Firmicutes* (up to 3%) biomass was observed compared to unmodified anode.^111^ Modification of the CC anode with PANI stimulated the participation of *Geobacter* sp. in the biofilm, while the simultaneous use of PDA with rGO on the CC anode caused that *Geobacter* sp. accounted for over 80% of the microorganisms identified in the biofilm. The anode modifications may act as a selective substrate for the growth of bacteria from the anolyte. Changes in the properties of the anode surface may also affect a transcriptomic profile of microorganisms in MFC; in the cells of microorganisms inhabiting the PDA/rGO modified anode, electrogenesis related to outer surface octaheme c-type cytochrome omcZ was highly expressed.^112^

##### 5.1.1.5. Composite anode materials

**Fig. 5:**
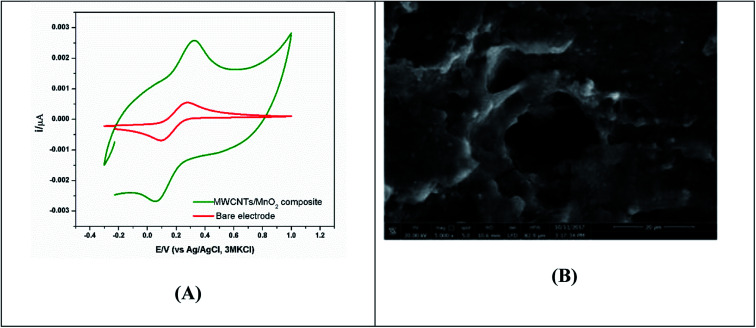
(A) Cyclic voltammetric characterizations of MnO_2_/MWCNTs nano-composite and bare electrode, (B) SEM images showing bioactive biofilm of *Enterobacter* sp. on the MnO_2_/MWCNTs nano-composite anode surface.^78^

Modification or implementation of nanostructured materials into or on traditional carbon structured electrode materials, such as 3D carbon black, carbon nanotubes (CNTs), porous carbon, graphene, and polyaniline (PANI), has also been highlighted as a potentially useful. Modification of anodes using metals or oxide-based nanocomposites influences the ohmic loss and result in improved bacterial cell attachment on the electrode surface. The newly fabricated anode, which consisted of a composite of iron (II, III) oxide (Fe_3_O_4_) and carbon nanotubes (CNT), produced a power density that reached up to 830 mW m^−2^. When Fe_3_O_4_ is attached to carbon nanotubes, it forms a multi-layered network that promotes bacterial growth and electron transfer.^113^ C/Hematite has been coated onto CC by the cost-effective and simple pyrolysis of ferrocene under atmospheric pressure. This procedure significantly improved the EET efficiency of *S. oneidensis* and has provided an approximately 6-fold higher current density in comparison to pristine CC anode in MFC.^114^ Recently, MnO_2_/MWCNTs nanocomposites were synthesized, characterized and utilized to support the growth of electrochemically active consortium of *Enterobacter* sp*.* Besides the formation of matured biofilm on its surface, MnO_2_/MWCNTs nanocomposite produced the highest electrical potential outputs (710 mV) combined with the highest power density (372 mW m^−2^), as shown in Fig. 5.^78^ Hydrothermal-assisted microwave dispersion and sonochemical synthesis were used to tin(iv) oxide (SnO_2_)-based monohybrids containing reduced graphene oxide (rGO) and carbon nanotubes (CNTs).^115,116^ During rGO/SnO_2_-CC based MFC operation, a power density of 1624 mW m^−2^ was achieved, which was 4.8-fold higher than that produced using a bare anode.^116^ The improved performance was primarily attributed to the increased specific surface area and higher biocompatibility of the hybrid composite which improve the bacterial adhesion and electron transfer efficiency.^117,118^

#### 5.1.2. The effect of scale-up of MFC system on the biofilm formation

MFCs have been investigated at multiple scales varying from a few microliters to 1000.0 L.^119–122^ Numerous factors such as diffusion of substrates/bio-products, electron transfer rate and biofilm thickness are involved in the regulation of the electron flow from the living microbial cells to the electrode surface (and *vice versa*). All these factors are greatly influenced by the scale, and size of the MFC being used. It was formerly demonstrated that biofilm formation was more preferred in microhabitats than in other niches.^123^ This is due to the fact that miniature systems provide higher productivity with shorter start-up times and lower substrate consumption.^124,125^ Their main disadvantage is that they have relatively low current outputs, which hinders them from being used in industrial scale. Large-scale MFCs must be composed of highly electroactive species for applications such as wastewater treatment and bioremediation. At the moment, miniaturized and liter-scaled MFCs are being investigated in tandem to screen electroactive species for large-scale applications.

#### 5.1.3. Effect of the type of membrane

In MFC system, the membrane governs the electro-neutrality between the two chambers, which permits the selective transfer of cations or anions *via* the anode and cathode. Proton exchange membranes (PEM) are the most commonly used membranes in MFCs because they allow protons to be transferred from the anode to the cathode.^126^ It was found that the type and surface area of the membrane had a significant impact on the power generation on MFCs.^127^ This is because the type of membrane used determines the type of ions transferred, which has a direct impact on the pH of the solution. For instance, when Nafion 117 was used, it was noticed that instead of proton pumping, other cations (such as Na^+^, K^+^, Ca^2+^, and Mg^2+^) present in the anolyte were transferred to the cathode, causing an increase in pH at the cathode and a subsequent reduction in pH at the anode. This pH variation affected the growth and community structure of the anodic biofilms.^128^

#### 5.1.4. Membrane biofouling and cost

Problems facing PEM use in MFC are biofouling and expensive cost. Biofouling of the membrane is an unwanted attachment of bacterial cells adhering to the membrane surface. It takes place initially *via* adhesion of organic or biological materials to the membrane and results in hindering proton transfer and increasing ohmic and charge transfer resistance. Strategies to prevent biofouling focus on: (1) preparation of antifouling composite membrane, (2) changing physical and chemical properties of existing membranes or (3) coating the membrane with anti-fouling agents.^129^ The main idea about preventing adhesion is to control the roughness, hydrophilicity and charge on the membrane surface. In terms of structure, a model membrane would be one that is smooth, negatively charged and highly hydrophilic.^130^ Several studies have reported on the anti-biofouling, for example, pre-treatment of Nafion 117 with 3% hydrogen peroxide and 0.5 M sulfuric acid resulted in less biofouling, and increased the power density to 186.7 mA W^−2^ as compared to only 20.9 mA W^−2^.^131^ Polyvinyl alcohol (PVA) has been reported as a suitable material for a smooth surface membrane, adding light expanded clay aggregates to the hydrophilic polyvinyl alcohol hydrogel (PEM-PVA-H-clay) has led to an increase in the proton conductivity with 2.87 fold as compared to that lacking clay.^132^ The addition of hydrophilic polydopamine coating on the membrane surface led to a reduction in the membrane biofouling with an increase in the direct electric current.^133^ While adding silver nanoparticles to polydopamine prevented PEM fouling due to its antimicrobial nature.^134^ Affordable PEM is also considered a bottleneck, a low cost activated carbon derived from coconut shell and blended with natural clay enhanced PEM hydration property and proton transfer which led to an increase in the coulombic efficiency of the MFC.^135^ In another report, ceramic membrane modified with rice husk ash exhibited high proton transfer and low oxygen diffusion,^136^ while sulfonated biochar-PVA composite showed an increase in the proton conductivity (32 fold) compared to Nafion membranes.^137^

### 5.2. Operation condition

During MFC operation, different physicochemical parameters may affect biofilm formation, and the overall performance of MFC. These parameters include pH, temperature, substrate, and ion concentration. This can be attributed to both bacterial and material surfaces being influenced by their surrounding environmental conditions.^138,139^

#### 5.2.1. Effect of pH

Effect of anodic pH on the performance of MFCs has been the main focus of many researchers.^140–143^ It was observed that the optimum pH for most MFC designs was restricted to neutral^7^ or near-neutral^6–8^ ranges. However, the operation of MFCs at extreme pH conditions was seldom reported.^142,144^ Though several factors (such as ion transfer, substrate oxidation, and oxygen reduction) are pH dependent during MFC operation, their effect can be circumvented by using appropriate catalysts, novel ion exchange membranes, and addition of buffering agents. At the same time, pH of the electrolyte has a direct influence on the growth and development of bacterial communities and their viability.^145^ This is because microorganisms are sensitive to their surrounding medium, and even the slightest variation in pH can cause alterations in cellular metabolism. However, unlike enzymatic reactions, bacteria can adapt themselves to varying external pH to carry out their metabolic reactions. Several physiological parameters such as the membrane potential, proton motive force, cytoplasmic, and electrochemical gradient are affected by the pH of the microenvironment and they ultimately influence the net production and consumption of protons.^145^ During MFC operation, especially when wastewater is used as a substrate, microorganisms are exposed to varying pH conditions at different time intervals due to the charge transfer between the anode and cathode.^141^ This change in pH did not only affect the power outputs of MFCs, but also led to a change in the microbial community.^145^ Apart from power outputs, due to the pH changes, the activity of anode-formed biofilm was inhibited, and thus the efficiency of MFCs for wastewater treatment was inhibited. Therefore, it is essential that the pH of the system is monitored periodically and maintained to obtain high power as well as high bio-degradation performance. Furthermore, sustaining an optimum pH for bacterial growth improves the robustness and longevity of biofilms. However, operating MFCs at constant pH by the addition of acid/alkali increases the cost of the process, which is undesirable. Thus, most studies were focused on maintaining the pH *via* the addition of a suitable buffer^146^ or *via* the use of anion exchange membranes.^147^ Though these studies have elaborated the role of pH during biofilm formation, further investigation is required to understand its effect on the microbial community structure and their interactions.

#### 5.2.2. Effect of temperature

Microbial biofilms are sensitive to temperature to optimize their growth and electrocatalytic activity.^148,149^ Depending on the temperature range in which they grow, bacteria can be divided into three groups, psychrophiles (<15 °C), mesophiles (15–40 °C), and thermophiles (>40 °C). Most of the MFC designs have been performed in mesophilic ranges,^147,150,151^ which suggest that most of the electroactive bacteria are functioning in mesophilic temperatures. However, certain psychrophiles and thermophiles have also been reported to be electroactive in nature.^151^ In MFCs with mixed cultures, temperature determines the nature and distribution of microbial communities as each species present in the consortia will have different optimum temperatures for growth.^150^ Furthermore, temperature variation leads to change in the kinetics and thermodynamics of the anodic reactions, which indirectly affect the growth of biofilms.^150^

#### 5.2.3. Effect of substrate concentration

The nature of the substrate and its concentration in the growth medium influence the dominance of one species over the other in a mixed microbial community.^152,153^ Different substrates, electron donors, ranging from simple sugars to complex carbohydrates, artificial, real wastewater, lignocellulosic biomass, and some of inorganic substrates, such as sulfide have been used in MFCs.^154^ A prominent shift in the prevailing dominant microbial community was observed with a change in the substrate concentration.^155^ This is due to the presence of different microorganisms rather than the electroactive bacteria present in the mixed consortia, which compete for the substrate consumption to gain energy. The substrate concentration, on the other hand, influences the power generated from the MFC system. In general, it was observed that high substrate concentration caused damaging effects to the biofilm. To achieve high coulombic efficiency, all electrons released from substrate oxidation should be channeled toward electricity generation. However, some of these electrons are lost, lowering the efficiency of the system. These intermediate compounds, if toxic to the growth of bacteria, can severely affect biofilm formation and current generation.

### 5.3. The biological parameters affecting biofilm formation

The biological factors that influence biofilm formation include the source and nature of inoculum, and growth rate. The majority of electroactive microbes tested to date have been found to be Gram-negative in nature.^156–158^ The change in cell wall composition between Gram-negative and Gram-positive bacteria is thought to cause a variation in cell surface charge, which influences the bacteria's electrogenic activity.^159^ Gram-positive bacteria have a peptidoglycan layer linked to teichoic acid, which gives them a high zeta potential, whereas Gram-negative bacteria have lipopolysaccharides, which give them a lower zeta potential than Gram-positive bacteria. As previously described, these bacterial surface charges interact with charges on the electrode surfaces, and thus is critical in deciding the sort of material to be utilized in the MFC system. Furthermore, bacterial biofilms can be created by a pure culture or a mixed culture.^160^ Consequently, the bigger the number of electroactive species present, the faster the substrate degrades and the more protons and electrons are produced. The outputs of MFCs can be significantly increased if all electrons released during substrate degradation are redirected for energy generation. When compared to pure culture biofilms, mixed culture biofilms were found to yield a higher flow of electrons.^161^ However, the bacterial community present in the mixed culture is inconsistent; making the reproducibility of such biofilm is debatable. The source of the bacterial inoculum is also important in determining the current biofilm's composition. Electroactive microorganisms are abundant in nature and can be found in a variety of environments.^162^ Therefore, the bacterial population that prevails in an MFC is determined by the source of the inoculum. It was found that mixed cultures use wastewater, anaerobic sludge, or river/marine sediments as the main source of MFC inoculum.^163^ These mixed cultures are usually favored when the main use is wastewater treatment. But, pure cultures (such as *Clostridium* sp.) have been used for selective applications, such as BOD biosensors, to achieve batch-to-batch repeatability of the signal output.^164^

## 6. Challenges of cathodic oxygen reduction reaction (ORR)

**Fig. 6:**
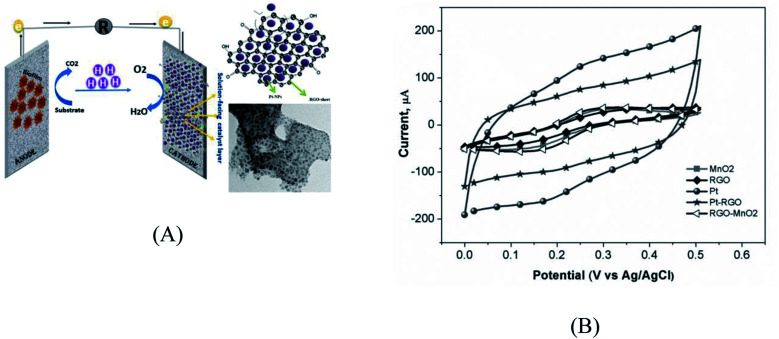
(A) Schematic representation showing the effect of nanocomposite on the ORR at the cathodic side. (B) Electrochemical catalytic activities of modified electrodes with the prepared nanostructures toward the reduction of dissolved oxygen in the voltammetric cells.^165^

At the cathodic chamber, oxygen is commonly used as the final electron acceptor because of its high oxidation potential. Many studies reported that the oxygen supply to the cathode compartment is energy consuming.^165^ While the atmospheric oxygen can be used directly by using an air cathode, contact difficulties in the cathode-air surface and the necessity for expensive catalysts are the disadvantages of oxygen utilization.^166^ The development of low-cost, high-performance nanocatalysts for the intrinsically slow oxygen reduction process (ORR) is a major roadblock in the commercialization of fuel cells for energy conversion. Recently, a lot of research has been directed towards developing Pd-based nanocatalysts with improved stability to use as Pt alternatives.^167^ Over the years, a wide range of investigations have been focused on the utility of Pd-based alloys during oxygen reduction. For example, Liu *et al.* developed a surfactant-based synthetic technique to synthesize Pd–Ni nanowires with uniform metallic elements dispersion.^168^ A previous study showed that the power density of Pt/rGO cathode based MFC was reduced by 5.8% compared to Pt/C cathode. Even though, the power densities for non-Pt cathode catalysts was reduced to 26.4%, 31.1%, and 48.2% for MnO_2_/rGO, rGO, and MnO_2_, respectively. Generally, the use of MFCs equipped with the Pt/rGO cathode, Pt/rGO was found to be a relatively cheaper catalyst that can be produced using a facile preparation method in MFC applications. This approach that shown in Fig. 6 should be encouraged, while the voltammetric technique is very efficient in the assessment or screening of new materials that might show catalytic activities towards the ORR.^165^

## 7. MFC scaling up issues

In order to treat large volumes of wastewater, and generate high current density, scaling up the MFC is essential. It has been demonstrated that as the dimension/configuration of MFC is enlarged, the power density decreases owing to the volumetric ohmic resistance increase and inactive reactor volume, resulting in low MFC power production performance.^169^ Integrating/collecting multiple small MFC systems to build a large stack for power generation is more realistic and efficient way to scale-up MFC systems than simply increasing the size of each reactor.^170^ So far, there have been a number of studies that have investigated at the performance of a scale-up MFC stack.^171,172^ Unfortunately, in some studies the reported power densities were still too low to make the MFC system comparable to traditional anaerobic treatment in terms of energy recovery. For instance Dekker *et al.*^173^ fabricated a 20 L stacked MFC fed by synthetic wastewater but due to the reversal voltage in some cell units under series electrical connection, the produced power density was very low and didn't exceed 11 W m^−3^. Hence, to improve the power output of MFC, the internal resistance which primarily includes kinetic, ohmic, and transportation resistance should be decreased as low as possible.^174^

## 8. Concluding remarks and future perspectives

MFCs are a novel suitable, eco-friendly alternative to produce energy through the oxidation of a variety of substrates; however, there are some challenges that need to be addressed to make the technology economically sound. The main prime hurdle is the active biofilm formation which is a critical key for MFC performance. Hence, it is important to understand electroactive biofilms in detail, the electron transfer mechanisms, and the factors affecting biofilm formation. In addition, this review article emphasizes on the importance of a feasible design for MFC scale-up. The majority of designs exhibit drawbacks such as high internal resistance, electrode spacing, exchange of anolyte and catholyte across PEM for scaling-up and long-term operation. Another challenge is to provide cost-effective electrode materials and PEM (if used) for MFCs. In previous decades, many conventional anode materials were introduced, but they have failed to meet the modern demands. An ideal anode would provide basic conductive properties and good microbe-electrode interface that lead to higher electron transfer rates and hence, high MFC performance. Among all the materials, the natural resources and their composite are good materials to fulfill the modern requirements while minimizing other disadvantages. One more obstacle is the choice of electroactive microbes because microbial surface charges interact with charges on the electrode surfaces, and thus is critical in deciding the type of material to be utilized in the MFC system. Indeed, more efforts are required to overcome these difficulties and enhance the overall performance of MFCs prior to industrial scale.

On the other hand, oxygen reduction electrocatalysis is very important for many applications including the microbial fuel cells. However, the cathodic oxygen reduction reaction requires the development of effective, and high performance electrocatalysts to facilitate power output in MFCs. Due to the high cost, replacing noble metal-based electrocatalysts with highly efficient and inexpensive nanomaterials for ORRs is very crucial for the practical application of these technologies. This constrain has been covered in a section of this review.

## Conflicts of interest

There are no conflicts to declare.

## Supplementary Material
